# Usefulness of daptomycin lock therapy in children with catheter-related bacteremia after failed vancomycin lock therapy

**DOI:** 10.1186/s12941-023-00604-z

**Published:** 2023-06-22

**Authors:** Celia Permuy, Jone Ruiz-Azcárate, Mercedes Sampedro, Cristina Jiménez, Fernando Baquero-Artigao, Cristina Calvo, Ana Méndez-Echevarría

**Affiliations:** 1 Department of Pharmacology, Hospital Universitario La Paz, Madrid, Spain; 2 Paediatric Infectious and Tropical Diseases Department, Hospital Universitario La Paz., Paseo de La Castellana 261, 28046 Madrid, Spain; 3 Translational Research Network in Pediatric Infectious Diseases (RITIP), Institute for Health Research IdiPAZ. CIBERINFEC, Instituto de Salud Carlos III, Madrid, Spain

**Keywords:** Daptomycin, Vancomycin, Catheter-related infections, Child, Biofilm

## Abstract

**Purpose:**

Catheter-related bacteremia (CRB) is a significant cause of morbidity, resource expenditure and prolonged hospital stays in patients with long-term catheters, whose numbers have increased considerably in recent years. Antibiotic lock therapy reaches high concentrations in the catheter, allowing good penetration into the biofilm, being vancomycin the most commonly used one in gram-positive infections. Several authors have recently reported the superior in vitro efficacy of daptomycin compared with vancomycin, especially for eradicating biofilms. Although there is some data on the use of daptomycin for antibiotic lock in animal models and adults, there are no data on its use in children.

**Methods:**

A descriptive study was conducted in a tertiary hospital, including patients younger than 16 years in whom daptomycin lock therapy was employed between 2018 and 2022.

**Results:**

We report three pediatric patients in whom CRB was confirmed on admission by paired blood cultures positive for CoNS sensitive to vancomycin, daptomycin and linezolid. All patients started vancomycin lock therapy and systemic antibiotic therapy with proven sensitivity for the isolated bacteria, without achieving negative blood cultures. Due to the persistence of positive cultures, vancomycin lock therapy was replaced by daptomycin, and blood cultures turned negative, with no relapses or need for catheter removal.

**Conclusion:**

The use of daptomycin lock therapy could be considered in children with CoNS catheter infection, especially when other antibiotic lock therapy had failed.

## Introduction

Catheter-related bacteremia (CRB) is a significant cause of morbidity, resource expenditure and prolonged hospital stays in intensive care units, oncohematology wards and in patients with long-term catheters, whose numbers have increased considerably in recent years [1].

Gram-positive bacteria are the main cause of CRB in children [2], can create biofilms, and are isolated in up to 77.5% of all pediatric cases [2], in 70% of children undergoing parenteral nutrition [3], and in 73% of children diagnosed with oncological disease [4]. Many cases of CRB caused by coagulase-negative staphylococcus (CoNS) do not require catheter removal and can be treated with antibiotic lock therapy [5]. In CRB caused by *S. aureus*, catheter removal is recommended [5, 6], especially if there are associated complications [2]. However, in certain children, achieving a new venous access can be challenging, and therefore, retaining the catheter could be crucial.

Antibiotic lock therapy reaches high concentrations in the device, allows good penetration into the biofilm, is compatible with anticoagulants, and has prolonged stability, low toxicity risk, few adverse effects, low resistance potential and a good cost-effectiveness balance [7]. The most commonly used antibiotic for antibiotic lock in gram-positive infections is vancomycin, with a 70% penetration rate in *S. aureus* and CoNS biofilms [8].

Several authors have recently reported the superior in vitro efficacy of daptomycin compared with vancomycin, especially for eradicating biofilms [9]. Although there are some data on the use of daptomycin for antibiotic lock in animal models and adults [10–12], there are no data on its use in children. We report on three pediatric patients in whom vancomycin lock therapy failed and for whom daptomycin lock therapy was effective in saving their devices.

## Material and methods

A descriptive study was conducted in a tertiary hospital and included all patients younger than 16 years in whom daptomycin lock therapy was employed between 2018 and 2022. The study retrospectively collected their clinical and microbiological data.

All participants had previously undergone vancomycin lock therapy (a solution of 5 mg/mL of vancomycin and 50 IU/mL of heparin) every 24 h. After these hours, the antibiotic was removed and the catheter was again locked.

Depending on the size of the catheter, 2 to 4 mL of this solution was administered [5]. For daptomycin lock, a solution with a concentration of 5 mg/mL of daptomycin and 100 IU/mL of heparin was prepared, administering a volume of 2–4 mL depending on the size of the catheter [13]. Daptomycin lock was also administered every 24 h. Reconstitution of the daptomycin vial was performed using Ringer’s lactate solution.

## Results

Three children with CRB who underwent daptomycin lock therapy were included in the study *(*Table 1*)* (median age, 2 years; range 2–15). All were admitted for suspected CRB and were carriers of a reservoir-type catheter. Case 1 had previously been admitted in the same year (5 months earlier) for CRB due to *S. epidermidis*. The patient was treated for 5 days with intravenous vancomycin and vancomycin lock therapy for 3 days, without catheter removal.

**Table 1:** Participants with central venous catheter infection who underwent antibiotic lock with daptomycin

	Case 1	Case 2	Case 3
Age (years)	2	15	2
Underlying disease	Hemophilia A	Hemophilia A	VII factor deficiency
Type of catheter	Port-A-Cath	Port-A-Cath	Port-A-Cath
Microorganism	*S. epidermidis*	*S. oralis*	*S. condimenti*
Systemic therapy before starting daptomycin	Vancomycin (40 mg/kg/day)	Amoxicillin—Clavulanic acid (100 mg/kg/day)	Vancomycin (40 mg/kg/day) and Rifampicin (20 mg/kg/day)
Previous antibiotic lock	Vancomycin	Vancomycin	Vancomycin
Duration with previous antibiotic lock (days)	3	2	7
Indication for antibiotic lock with daptomycin	Persistent positives blood cultures	Persistent positives blood cultures and fever	Persistent positives blood cultures
Systemic therapy associated with antibiotic lock	Vancomycin (40 mg/kg/day)	Amoxicillin—Clavulanic acid (100 mg/kg/day)	Daptomycin (10 mg/kg/day)
Blood culture since starting daptomycin sealing (days)	2	1	5
Duration of antibiotic lock with daptomycin (days)	16	6	15
Catheter removal	No	No	No
Relapse	No	No	No
Total hospital stay (days)	9	12	71*
Hospital stay since the start of the antibiotic lock with daptomycin (days)	7	3	60*
Adverse effects	No	No	No

*Admission was prolonged due to complications of the patient’s underlying disease (bleeding in a patient with factor VII deficiency), which was not due to bacteremia

In all cases, CRB was confirmed on admission by paired blood cultures positive for CoNS sensitive to vancomycin, daptomycin and linezolid. All patients started vancomycin lock therapy and systemic antibiotic therapy with proven sensitivity for the isolated bacteria, without achieving negative blood cultures (median duration, 3 days; range, 2–7 days). Due to the persistence of positive blood cultures obtained from the central line, and the persistence of fever in patient 2, vancomycin lock therapy was replaced by daptomycin, and blood cultures turned negative after 2 days (range, 1–5 days). The patients remained with lock therapy with daptomycin for a median of 15 days (range, 6–16 days) and with intravenous antibiotic therapy for 7 days (range, 2–14 days), with no relapses or need for catheter removal.

## Discussion

Daptomycin is a cyclic lipopeptide with potent and rapid bactericidal effect both in vivo and in vitro studies [12] and is active against gram-positive bacteria, including vancomycin-resistant *S. aureus* [14]. Daptomycin acts by binding to the cytoplasmic membrane, producing an inhibition of DNA proteins, and has high biofilm penetration for *S. epidermidis.*

Daptomycin has recently shown superior effectiveness over vancomycin in reducing bacterial loads and in activity against biofilms [14, 15] and could therefore play a role in treating infections refractory to conventional antibiotic lock therapy [11], as we have observed.

In randomized, double-blind, in vitro, experimental animal studies, daptomycin showed superior in vitro activity against biofilms than vancomycin [9, 10, 16]. Most such studies performed with human patients are descriptive and retrospective and include adult populations [8, 10–12]. *Del Pozo *et al*.* were the first to evaluate the in vivo activity of daptomycin lock in adults, with apparently beneficial results in catheter preservation [16]. Recently, Blanco-Di Matteo et al. reported that daptomycin lock effectively eradicates CoNS catheter colonization, negativizing blood cultures obtained through the catheters, a finding that shows the superior diffusion of daptomycin inside staphylococcal biofilms compared with vancomycin [15].

There are studies on the use of systemic daptomycin for treating gram-positive bacterial infections in children, although there is currently scarce evidence on its use as a lock therapy. *Namtu *et al*.* retrospectively described the effectiveness of systemic daptomycin in children diagnosed with these infections. The authors also performed a catheter lock in 28% of their patients, in addition to systemic therapy with daptomycin, reporting a 73% rate of catheter preservation [14].

On the microbiological side, in vitro daptomycin can rapidly clear the pathogen [13], which is clinically important, especially for immunocompromised or severely ill patients, allowing for shorter therapies, with considerable benefits in terms of quality of life and reduced costs [10].

Daptomycin also has fewer drug interactions than glycopeptides and linezolid, with less renal and bone marrow toxicity [14].

In our study, the lock solution was prepared with Ringer’s lactate solution. Given that daptomycin is a calcium-dependent antibiotic, a study found that bacterial clearance from the catheter is higher when the solution is prepared with Ringer’s lactate solution (which contains calcium) instead of saline [13]. Numerous similar studies have not specified the solution’s concentration; however, those that did reported good responses with concentrations of 5 mg/mL [10, 12, 15], which is the same concentration as that used in our study.

A clear definition of antibiotic lock failure has not been established and many patients presenting with multiple positive catheter-drawn blood cultures need prolonged lock therapy before achieving the catheter salvage. Thus, prolonged vancomycin lock therapy might have also obtained catheters´ salvage in our patients. Nevertheless, daptomycin is a good option for the antibiotic lock of catheters and could be more effective than vancomycin in reducing bacterial load and bacterial biofilms, with fewer adverse effects and lower toxicity. Daptomycin may be considered, especially if other antibiotic lock therapies have failed. Randomized studies focusing on the safety and efficacy of daptomycin are needed to confirm and expand the appropriate use of daptomycin lock therapy in children.

## Data Availability

All clinically relevant data have been made available within the manuscript. For data protection reasons, no further clinical data can be made available.

## References

[1] Niedner MF (2010). The harder you look, the more you find: catheter-associated bloodstream infection surveillance variability. Am J Infect Control.

[2] Broudic M, Bodet LM, Dumont R, Joram N, Jacqmarcq O, Caillon J (2020). A 1-year survey of catheter-related infections in a pediatric university hospital: a prospective study. Arch Pediatr.

[3] Al Lawati TT, Al Jamie A, Al MN (2017). Central line associated sepsis in children receiving parenteral nutrition in Oman. J Infect Public Health.

[4] Gowin E, Świątek-Kościelna B, Mańkowski P, Januszkiewicz-Lewandowska D (2020). The profile of microorganisms responsible for port-related bacteremia in pediatric hemato-oncological patients. Cancer Control.

[5] Meímel LA, Allon M, Bouza E, Cíaven DE, Flynn P, O'Gíady NP (2009). Clinical practice guidelines for the diagnosis and management of intravascular catheter-related infection. Clin Infect Dis.

[6] Hogan S, Zapotoczna M, Stevens NT, Humphreys H, OGara JP, ONeill E (2016). *In vitro* approach for identification of the most effective agents for antimicrobial lock therapy in the treatment of intravascular catheter-related infections caused by the *Staphylococcus aureus*. Antimicr Agents Chemother.

[7] Justo JA, Bookstaver PB (2014). Antibiotic lock therapy: review of technique and logistical challenges. Infect Drug Resist.

[8] Alonso B, Fernández-Cruz A, Díaz M, Sánchez-Carrillo C, Martín-Rabadán P, Bouza E (2020). Can vancomycin lock therapy extend the retention time of infected long-term catheters?. APMIS.

[9] Basas J, Palau M, Ratia C, Del Pozo JL, Martín-Gómez MT, Gomis X (2018). High-dose daptomycin is effective as an antibiotic lock therapy in a rabbit model of staphylococcus epidermidis catheter-related infection. Antimicrob Agents Chemother.

[10] Vassallo M, Genillier PL, Dunais B, Kaphan R, Saudes L, Duval Y (2017). Short-course daptomycin lock and systemic therapy for catheter-related bloodstream infections: a retrospective cohort study in cancer patients with surgically implanted devices. J Chemother.

[11] Tatarelli P, Parisini A, Del Bono V, Mikulska M, Viscoli C (2015). Efficacy of daptomycin lock therapy in the treatment of bloodstream infections related to long-term catheter. Infection.

[12] Pozo JL, Rodil R, Aguinaga A, Yuste JR, Bustos C, Montero A (2012). Daptomycin lock therapy for grampositive long-term catheter-related bloodstream infections. Int J Clin Pract.

[13] Van Praag A, Li T, Zhang S, Arya A, Chen L, Zhang X (2011). Daptomycin antibiotic lock therapy in a rat model of staphylococcal central venous catheter biofilm infections. Antimicrob Agents Chemother.

[14] Namtu KC, Crain JC, Messina AF, Dumois JA, Berman DM (2017). Clinical experience with daptomycin in pediatrics. Pharmacotherapy.

[15] Blanco-Di Matteo A, Garcia-Fernandez N, Aguinaga Pérez A, Carmona-Torre F, Oteiza AC, Leiva J (2023). In vivo effectiveness of several antimicrobial locks to eradicate intravascular catheter coagulase-negative staphylococci biofilms. Antimicrob Agents Chemother.

[16] Meije Y, Almirante B, del Pozo JL (2014). Daptomycin is effective as antibiotic- lock therapy in a model of Staphylococcus aureus catheter-related infection. J Infect.

